# Synthesis and Characterization of Hexagonal Boron Nitride as a Gate Dielectric

**DOI:** 10.1038/srep30449

**Published:** 2016-07-26

**Authors:** Sung Kyu Jang, Jiyoun Youn, Young Jae Song, Sungjoo Lee

**Affiliations:** 1 SKKU Advanced Institute of Nano Technology (SAINT), Sungkyunkwan University (SKKU), Suwon, Gyeonggi-do 440-746, South Korea; 2 Department of Physics, Sungkyunkwan University (SKKU), Suwon, Gyeonggi-do 440-746, South Korea; 3 Center for Integrated Nanostructure Physics, Institute for Basic Science (IBS), Suwon 16419 Republic of Korea; 4 College of Information and Communication Engineering, Sungkyunkwan University (SKKU), Suwon, Gyeonggi-do 440-746, South Korea; 5 Center for Human Interface Nanotechnology (HINT), Sungkyunkwan University (SKKU), Suwon, Gyeonggi-do 440-746, South Korea

## Abstract

Two different growth modes of large-area hexagonal boron nitride (h-BN) film, a conventional chemical vapor deposition (CVD) growth mode and a high-pressure CVD growth mode, were compared as a function of the precursor partial pressure. Conventional self-limited CVD growth was obtained below a critical partial pressure of the borazine precursor, whereas a thick h-BN layer (thicker than a critical thickness of 10 nm) was grown beyond a critical partial pressure. An interesting coincidence of a critical thickness of 10 nm was identified in both the CVD growth behavior and in the breakdown electric field strength and leakage current mechanism, indicating that the electrical properties of the CVD h-BN film depended significantly on the film growth mode and the resultant film quality.

Metallic, semiconducting, or insulating two-dimensional (2D) materials have been extensively investigated in recent decades, both with respect to their fundamental properties and their device fabrication techniques, due to the utility of these 2D materials in a wide range of potential applications. Graphene was identified as the first truly 2D material with partial metallic properties^1^,^2^,^3^. Subsequent studies of the transition metal dichalcogenides (TMD)^4^,^5^,^6^ and black phosphorus^7^,^8^ identified additional 2D semiconducting candidate materials for use in electronic and photonic device applications. Hexagonal boron nitride (h-BN) has been suggested as an ideal insulating template for 2D devices due to its excellent properties. h-BN has a high mechanical hardness and resilience due to its strong covalent bonding^9^, a wide direct bandgap (5.9–6.1 eV)^10^, a small lattice mismatch (1.7%)^11^ with graphene, and a high thermal conductivity^12^,^13^. h-BN has been employed in a wide range of devices as a transparent membrane^14^, encapsulation material^15^,^16^, tunneling barrier^17^,^18^, and dielectric substrate^19^. For instance, graphene field effect transistors integrated with an h-BN dielectric layer exhibited enhanced mobilities and current on-off ratios, compared to the values obtained from graphene devices stacked on other substrates^19^,^20^. The absence of dangling bonds or trapped charges, in addition to the atomic flatness of the h-BN surface, significantly enhanced the film performance when integrated with MoS_2_^21^,^22^ and other 2D materials^23^,^24^,^25^,^26^,^27^,^28^ However, high-performance insulating 2D materials for use in industrial applications have not yet been identified due to the lack of approaches for synthesizing large-area h-BN films with good thickness control and appropriate dielectric properties. Mechanical exfoliation has been widely used to produce high-quality h-BN, but this approach is strictly limited to the fabrication of small-scale devices. Exfoliation produces flakes of limited size, inconsistent yield, and variable number of h-BN layers. Chemical vapor deposition (CVD), by contrast, offers a popular route to synthesizing large-area h-BN films with easy control over the number of layers. The large-area and high-quality CVD growth of h-BN, therefore, is extremely important as a mechanical and chemical protection layer with flexible and bendable properties for industrial applications of graphene to electric devices. Although CVD growth using a transition metal catalyst has been reported for large-scale synthesis^29^,^30^,^31^,^32^, only few studies have described the successful growth of high-quality thick h-BN films. To apply h-BN films for the electronic devices, we need to control the thickness of CVD h-BN films from few nm to 100’s nm in large-scale. The catalytic effect in the conventional CVD for h-BN is limited up to ∼10 nm thickness, so it has been not possible to get large-area h-BN films of layered structures thick enough for different applications. The characteristics of highly crystalline CVD-grown h-BN dielectric layers as a function of the layer thickness have not yet been characterized. Thus successful synthesis of these layers and a comprehensive understanding of the thickness-dependent h-BN film properties as gate dielectric materials would lay the foundation for their use in future graphene-based nanoelectronic devices.

In this work, we developed a new CVD mode (high-pressure CVD growth) for thickness-controlled CVD growth of h-BN as an insulating 2D materials, which cannot be done by the conventional CVD growth. The thickness of a h-BN film grown on a Cu foil could be controlled by adjusting the partial pressure of the precursor and the low-pressure CVD process time. At partial pressures exceeding a certain critical value (17 mTorr in this work), the h-BN thickness increased linearly as a function of the growth time, up to a thickness of 100 nm. The thick h-BN layers were achieved by introducing new high-pressure CVD conditions that overcame the catalytic limits of h-BN growth on Cu. The atomic and chemical structures of the thick h-BN layers were characterized by optical microscopy (OM), atomic force microscopy (AFM), Kelvin probe force microscopy (KPFM), UV photospectrometry, Raman spectroscopy, scanning electron microscopy (SEM), and transmission electron microscopy (TEM). The electrical properties of the thin and thick h-BN films were systematically investigated in the context of metal-insulator-metal (MIM) and metal-insulator-semiconductor (MIS) capacitors in an effort to optimize the insulating properties of these films for use in electronic device applications. Excellent dielectric (a high breakdown field and a low leakage current) and interface properties, with low interface state densities, were obtained. Our results pave a path toward realizing 2D dielectric materials for use in future nanoscaled device applications.

## Results and Discussion

### CVD Synthesis and two different growth mode of h-BN

**Figure 1:**
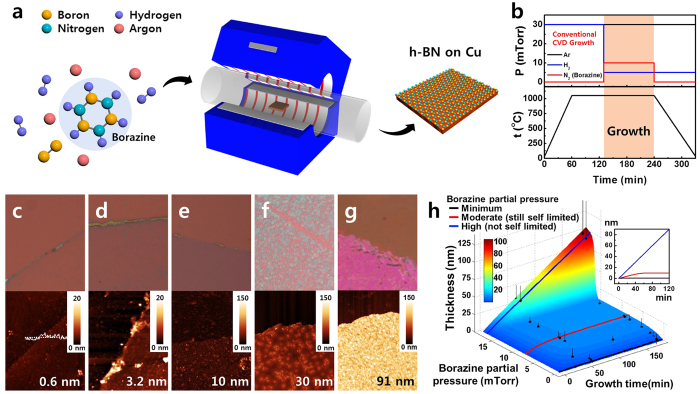
(**a**) Schematic diagram of the low-pressure CVD system used for h-BN growth. (**b**) Time-dependent parameter program for the h-BN growth, including the temperature profile and partial pressures. (**c**–**g**) Optical microscopy and corresponding AFM images of the h-BN films with different thicknesses. Each film was grown on a Cu foil and transferred onto 285 nm thick SiO_2_/Si substrates. The thicknesses of the h-BN films in (**c**–**g**) were 0.6, 3.2, 10, 30, and 91 nm, respectively. (**h**) A three dimensional plot of the h-BN thickness (z-axis) as a function of growth time (x-axis) and partial pressure of precursor (y-axis). The black dots represent the averaged thickness for corresponding growth time and partial pressure. The standard error of each data point represented by the vertical error bar is enlarged tenfold to visualize. The each interpolated line of blue, red and black in the figure is color-coded for different growth conditions of the high-, the moderate- and the minimum- partial pressure of borazine respectively. For better understanding, the inset of a 2D plot is added.

The growth of h-BN films using borazine (B_3_N_3_H_6_) gas as a precursor was carried out in a typical hot-wall furnace under low-pressure conditions, as described schematically in Fig. 1a. Prior to the CVD process, a wet and a dry pre-treatment were applied to the Cu foils to ensure the fabrication of highly crystalline h-BN. The Cu foil (Alfa Aesar) was polished with diluted copper etchant (Transene, type CE-100) to form a smooth surface, followed by rinsing with hydrofluoric acid solution (20%) to clean any remaining residue. After this wet treatment, the Cu foil was placed in the center of a heating zone and heated to 1050 °C for 2 hours under a dry gas flow of mixed Ar and H_2_ to improve the Cu grains^33^. Once the dry treatment of the Cu foils in the furnace had been finished, CVD growth of h-BN was carried out immediately over 1 hour at 1050 °C using borazine. The gas growth conditions are described in detailed in Fig. 1b. The borazine gas was supplied by flowing a nitrogen carrier gas through the bubbler of liquid borazine. The carrier gas flow rate was tuned between 0.1 and 20 sccm using two mass flow controllers at the inlet and outlet, holding the bubbler temperature at −15 °C to maintain a constant vapor pressure of borazine. After film growth, the furnace was slowly cooled to room temperature under an Ar/H_2_ flow.

It should be noted that the thickness of the h-BN film grown on the Cu foil could be tuned to yield films with a thickness of an atomic monolayer up to 100 nm by appropriately adjusting the growth time and partial pressure of borazine. Figure 1c–g show optical microscopy images (top) and the corresponding AFM images (bottom) of h-BN films transferred onto 285 nm SiO_2_/Si substrates. The growth of h-BN films (Fig. 1c–e) in a furnace was self-limited at typical pressures, whereas the growth (Fig. 1g) was not self-limited at higher pressures. As summarized in the three dimensional plot with growth time (x-axis), partial pressure (y-axis) and the film thickness (z-axis) in Fig. 1h, h-BN could be controllably grown to form atomic monolayers or 100 nm thick films, depending on the growth conditions: conventional CVD growth mode or high-pressure CVD growth mode. In the conventional CVD growth mode, the growth rate and the self-limited thickness were controlled and limited by the catalytic reaction at the surface^34^. A continuous atomic monolayer of h-BN, therefore, could be obtained by using 0.2 sccm borazine in the carrier gas (N_2_) during conventional CVD growth (black curve in Fig. 1h)^35^ The thickness of the h-BN films reached saturation at a critical thickness (10 nm in our work, red curve in Fig. 1h) after sufficient growth time under conventional CVD growth conditions, which could be explained in terms of the catalytic transparency of h-BN on Cu^36^. This critical thickness is consistent with previous reports^37^, in which BN films could be grown up to 10 nm thick on a graphene/h-BN/Cu foil. CVD growth under much higher pressures in the furnace, however, significantly increased the h-BN film thickness up to 100 nm (blue curve in Fig. 1h). These results indicate that a new CVD growth mode was in operation during the production of the thick h-BN films (>10 nm) at high partial pressures of borazine in the furnace.

### Surface and structure Characterization of the h-BN Films grown with different growth modes

**Figure 2:**
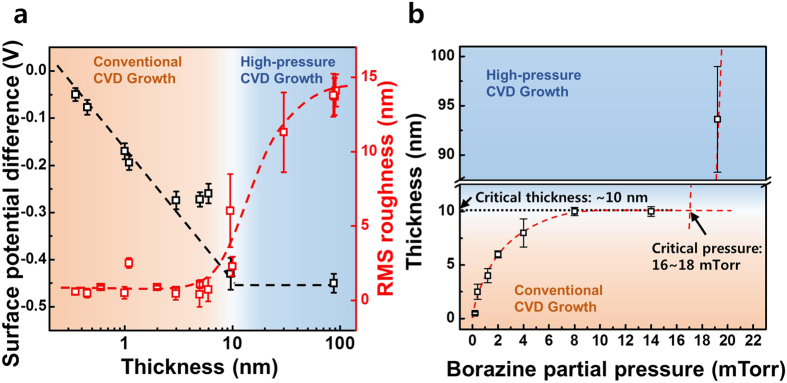
(**a**) The surface potential differences (black color) and RMS roughness values (red color) of the h-BN surfaces on SiO_2_ substrates as a function of the h-BN thickness. (**b**) A plot of film thickness after 3 hours growth with respect to the borazine partial pressure. A discontinuous first-order transition at the critical pressure (16∼18 mTorr) was observed.

Figure 2a summarizes the surface potentials and surface roughnesses of h-BN films of various thicknesses grown on SiO_2_ substrates. The self-limited thickness depended closely on the catalytic properties of the growth surface. The surface potentials of the h-BN films were directly measured using Kelvin probe force microscopy (KPFM) while simultaneously measuring the topologies using AFM. A maximal critical thickness of 10 nm was observed, as expected, and the surface potential obtained under conventional CVD growth conditions (thickness <10 nm) decreased linearly and reached saturation at 10 nm, as reported previously^36^. In the high-pressure CVD growth regime, very thick h-BN films (>10 nm thick) could be grown that could not be grown under conventional CVD conditions. Although the thick h-BN films were more than 10 nm thick when grown in the high-pressure CVD growth mode, the surface potential did not differ significantly from the value obtained from a 10 nm thick h-BN film grown under conventional CVD conditions. These results indicate that the conventional CVD growth mode dominated during growth up to a thickness of 10 nm, and the thicker h-BN films grew in a distinct regime, the high-pressure CVD growth mode. Interestingly, an abrupt change in the surface morphology and roughness was observed as the thickness of h-BN films increased as shown in Fig. 2a (red color). An RMS roughness of 0.4 nm was obtained from the atomic monolayer, and this value remained constant below the critical thickness (10 nm), the roughness began to increase near the critical thickness and then reached saturation above 10 nm. The catalytic growth of h-BN was not possible at thicknesses exceeding 10 nm. SEM images of the h-BN films (Supplementary Information Figure S1) revealed rough surface morphologies with island structures on the top surface. Similar island structures have been observed previously during the CVD growth of a thick top BN layer on graphene/h-BN/Cu substrates^37^. Figure 2b shows the film thickness as a function of the partial pressure of borazine after 3 hours of growth. As expected, the thickness of the h-BN film increased and saturated at 10 nm, if the partial pressure of borazine was less than a critical pressure (∼17 mTorr in our particular experiment environment). A discontinuous first-order transition at the critical pressure was observed in the curve of the thickness as a function of the partial pressure of borazine. This plot confirmed that a new growth mode (high-pressure CVD growth mode), rather than the conventional CVD growth mode, was introduced above the critical pressures. The mechanism underlying the abrupt change in the growth mechanism with respect to the partial pressure of the precursor is not yet completely understood. Further works are required to understand the growth mechanism. The film thickness under the high-pressure CVD growth mode depended linearly on the growth time, as demonstrated in a previous report of the controlled CVD growth of high-quality h-BN on Ni foils^32^.

**Figure 3:**
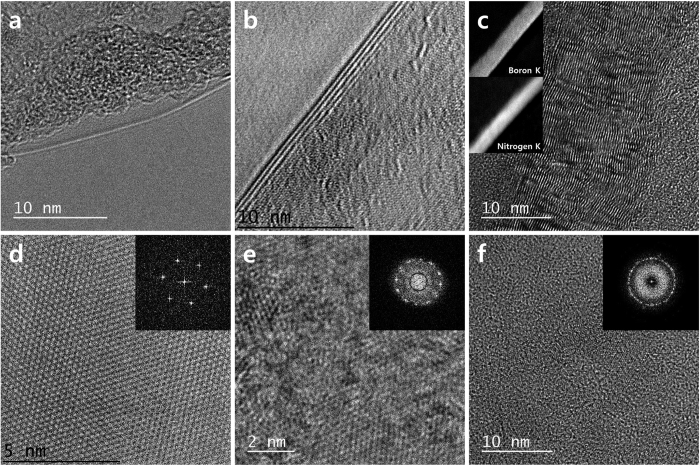
TEM images of the folded edges of (**a**) atomic monolayers and (**b**) 4-layer h-BN films. (**c**) Cross-sectional TEM image of a 20 nm thick h-BN film, prepared using FIB processing. The insets show the elemental EELS mappings. (**d**–**f**) In-plane high-resolution TEM images of each h-BN film shown in figure (**a**–**c**). The insets show the corresponding FFT images.

Figure 3a,b show the folded edge of a thin h-BN film (monolayer or 4 layers, respectively) grown using the conventional CVD growth mode. The layered structures may be clearly seen, and the interlayer distance was measured to be 3.5 Å, consistent with previous reports^30^,^38^. Figure 3c shows a cross-sectional TEM image of a thick h-BN film grown using the high-pressure CVD growth mode. As this thick h-BN film was mechanically robust, the film could be subjected to focused ion beam (FIB) milling. In this image, more than 70 h-BN layers may be counted. Electron energy-loss spectroscopy (EELS) measurements were carried out to examine the chemical composition of the film. The EELS spectrum (Supplementary Information Figure S2) revealed two visible edges located at 185 and 405 eV, corresponding to K-shell ionization of B and N respectively, indicating that the h-BN film was constructed from sp^2^-hybridized bonds^39^,^40^. The insets in Fig. 3c show the elemental mappings of cross-sections of the thick h-BN film. Boron and nitrogen may be clearly observed. Figure 3d–f show in-plane high-resolution TEM images of each h-BN film shown in Fig. 3a–c, and the insets show the Fast Fourier transform (FFT) data. As the number of layers increased, the six-fold symmetric peaks indicated the presence of extensive rotation for a N-N intralayer distance of 2.5 Å^41^. This result indicated the presence of rotational stacking faults among the h-BN layers or polycrystalline in-plane structures in the thicker h-BN films. The layered growth of a honeycomb lattice with the expected chemical composition was confirmed by TEM, FFT, and EELS mapping, revealing the high crystallinity and quality of the thin and thick h-BN films.

The optical band gap was investigated by UV-visible absorption spectroscopy (Supplementary Information Figure S3).The band gap is in the range of ∼6 eV to ∼5.6 eV, which shows a good agreement with the previous reports^31^. These structural and chemical configurations also could be supported by Raman spectroscopy and XPS measurements as shown in Supplementary Information Figure S4.

### Electrical property of CVD h-BN as a gate dielectric

**Figure 4:**
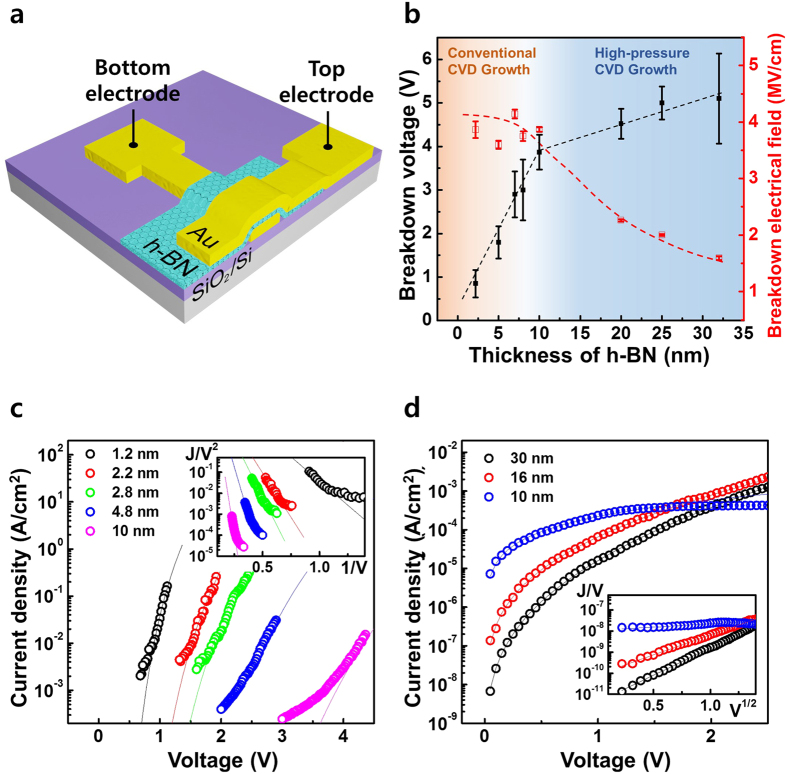
(**a**) A schematic diagram of the metal / h-BN / metal (MIM) capacitors fabricated on a SiO_2_/Si substrate. (**b**) The breakdown characteristics as a function of the h-BN film thickness. (**c**) Typical J-V characteristics of a MIM capacitor, described by the field-assisted tunneling model. The h-BN thickness range was less than 10 nm. The inset shows an FN tunneling plot (J/V^2^ versus 1/V). (**d**) Typical J-V characteristics of a MIM capacitor more than 10 nm thick, and the trap-assisted transport model. The inset shows the PF emission plot (J/V versus V^1/2^).

The dielectric properties of the CVD h-BN films with different thicknesses were measured by fabricating metal/h-BN/metal (MIM) capacitors and measuring their capacitance–voltage (C-V) and current–voltage (I-V) characteristics. Figure 4a shows a schematic diagram of a metal/h-BN/metal capacitor. The bottom electrode (Au) was fabricated using photolithography, gold deposition, and a lift-off process. The h-BN films of various thicknesses were transferred onto a substrate prepared with a bottom electrode. The same electrode preparation process was applied to form the top electrode. The contact area was 5*μm* × 5*μm*. The C-V and I-V characteristics were measured using a semiconductor parameter analyzer (Keithley 4200-SCS). The dielectric constant (*ε*_*r*_) was estimated from the measured MIM capacitance and the physical thickness, measured by AFM, was 3–4, regardless of the thickness of the h-BN film (Supplementary Information Figure S5). Figure 4b plots the breakdown voltage and field at which a catastrophic increase in current through the MIM capacitor was observed, as a function of the h-BN thickness. An h-BN film with a thickness of less than 10 nm, grown via the catalytic reaction as discussed above, displayed a breakdown voltage that increased linearly as the h-BN thickness increased, while the breakdown electric field remained above 4 MV/cm. By contrast, a significant decrease in the breakdown electric field was observed in h-BN films more than over 10 nm thick. This value reached 2.4 MV/cm at a thickness of 20 nm, similar to the values reported previously^42^. The leakage current densities at the two metal electrodes and through the h-BN layers of different thicknesses were investigated as a function of voltage, as plotted in Fig. 4c (<10 nm h-BN) and Fig. 4d (>10 nm h-BN). As shown in Fig. 4c, the measured leakage currents (circle symbol) of the thin h-BN films agreed well with the Fowler–Nordheim (FN) tunneling current model (line), indicating that FN tunneling provided the dominant carrier transport mechanism in the thin h-BN films. As the h-BN thickness increased, agreement with the FN model decreased, suggesting that other transport mechanisms applied. The inset in Fig. 4c shows the FN plot using the following equation^43^,^44^:


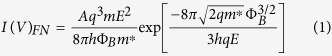


where *A*, *q*, *m*, Φ_*B*_, *E* and *h* are the effective area, electron charge, free electron mass, barrier height, electrical field, and Plank’s constant respectively. The electron effective mass m^*^ is 0.26 *m* in h-BN^41^. The linear slope of the FN plot was used to estimate the barrier between the metal and h-BN: 2.8–2.9 eV. Figure 4d shows the measured I-V curve (symbols) and the Poole–Frenkel (PF) emission model (line) for the thicker h-BN films. Almost complete agreement between the experimental results and theoretical model suggested that a trap-assisted PF emission mechanism constituted the dominant transport mechanism for the leakage current in the thick h-BN films. The inset in Fig. 4d shows the PF plot using the following equation^45^:


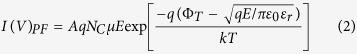


where *N*_*c*_, *μ*, and Φ_*t*_ the density of states in the conduction band, electronic mobility in the oxide and the trap energy level in the h-BN, respectively. The other notations are the same as defined before. The trap ionization energy, Φ_*t*_, was estimated from this curve as 0.3 eV below the conduction band edge of h-BN. Interestingly, the critical thickness of 10 nm was identified in the CVD growth behavior (Fig. 2a,b), in the electric field strength breakdown (Fig. 4b), and in the leakage current mechanism (Fig. 4c,d). The existence of a common threshold indicates that the electrical properties of the CVD h-BN film depended significantly on the film growth mode and the resultant film quality.

**Figure 5:**
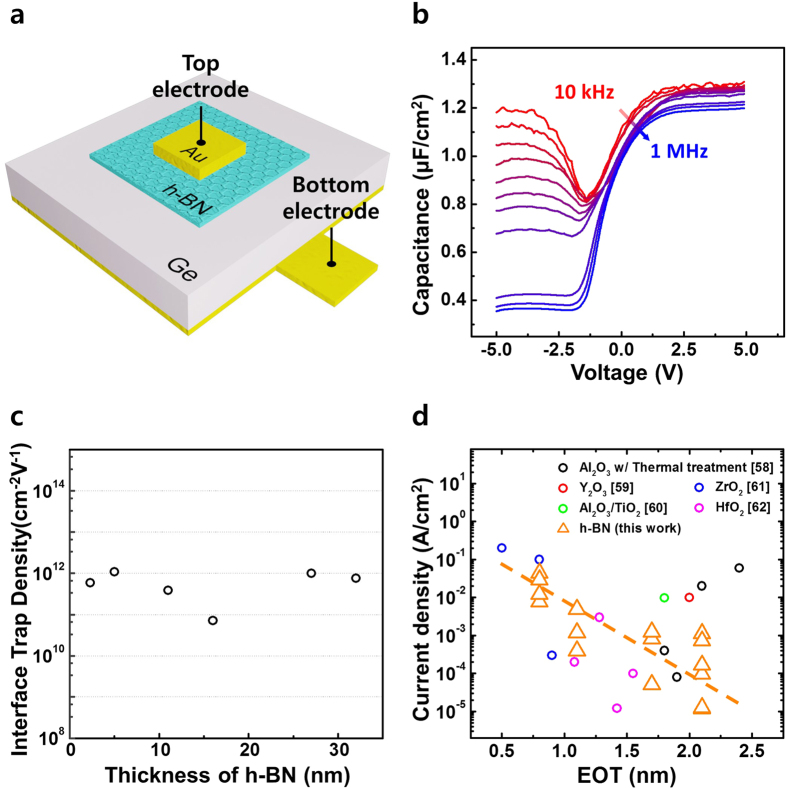
(**a**) Schematic diagram of the metal/h-BN/Ge (MIS) capacitor. (**b**) Representative C-V characteristics, C-V, at various frequencies. (**c**) The interface trap densities (D_*it*_) of h-BN MIS capacitors of different thicknesses, calculated using the high-low frequency method. (**d**) The leakage current densities of the MIS capacitors at V_*g*_ = −1 V, as a function of the EOT.

The electrical properties of a metal/h-BN/Ge MIS capacitor were characterized to investigate the properties and quality of the h-BN gate dielectric on a Ge substrate. MIS capacitors were fabricated by transferring the CVD-grown h-BN films onto a Ge substrate. The metallic electrodes were defined by photolithography, followed by Au (50 nm) evaporation and a lift-off process. Ge was selected as the substrate material because its low effective mass and high carrier mobility, which overcome some of the fundamental limitations on Si-based devices, renders it useful in future electronics^46^,^47^,^48^,^49^. Figure 5a shows a schematic diagram of a metal/h-BN/Ge MIS capacitor. The contact area was 20*μm* × 20*μm*. Figure 5b shows the typical C-V characteristics of the MIS capacitors. The h-BN layer was 11 nm thick. Over a measurement frequency range (10 kHz–1 MHz), the flat band voltage did not shift and no bumps in the C-V curves were observed. The interface trap density, *D*_*it*_, between the h-BN dielectric layer and the Ge substrate were evaluated by comparing the low- and high-frequency C-V characteristics using the following equation^50^:


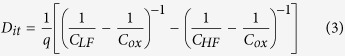


where *C*_*LF*_ is the capacitance measured at low frequencies, *C*_*HF*_ is the capacitance measured at high frequencies. Figure 5c shows the interface trap density as a function of the h-BN film thickness. The estimated *D*_*it*_ values lay in the range 10^11^–10^12^ *cm*^−2^ *eV*^−1^. Similar results were obtained using the Terman method^51^. Previous studies of Ge MOSFETs revealed that despite the high carrier mobilities obtained from Ge transistors, Ge-MOSFETs provide a poor interface quality, with a high density of interface traps or mid-gap bulk semiconductor traps, which produce a high leakage current and poor sub-threshold characteristics^52^. The lack of sufficiently stable oxides of Ge, unlike Si has hampered materials research in this area. Although recent progress in the development of high-K dielectrics has reopened the possibility of realizing CMOS devices on Ge, the reported density of the interface states in high-k/Ge systems tends to fall in the range 10^12^–10^13^ *cm*^−2^ *eV*^−1 ^53,54,55. Reactions at the interface, such as Ge diffusion from the substrate into the high-K dielectric^46^,^56^ or metal diffusion into the Ge substrate^57^, were reported as possible reasons for the poor interface quality. The values of *D*_*it*_ obtained from the Ge MIS capacitors integrated with CVD-grown h-BN films of various thicknesses indicated that a high-quality h-BN/Ge interface could be prepared using a low-temperature process for transferring the two-dimensional h-BN films onto a Ge substrate, which is helpful to suppress both a reaction between Ge and the dielectric materials and a formation of chemically unstable GeO or GeO_2_ surface layer. The interface quality could be further improved by optimizing the transfer process and minimizing impurities. Figure 5d shows the gate leakage current densities of the h-BN/Ge MIS capacitors as a function of the equivalent oxide thickness (EOT). Previous studies of high-K/Ge capacitors^58^,^59^,^60^,^61^,^62^ used different interlayer passivation processes between the high-K material and Ge. The results of these previous studies are included in the figure for comparison purposes. Our results revealed that comparable or suppressed leakage currents could be achieved from h-BN/Ge capacitors without introducing a Ge passivation layer, providing an EOT of 0.8–2.5 nm. These results demonstrated that the layer-controlled CVD-grown h-BN could be used as a dielectric material in scaled high-speed and low-power device applications.

## Conclusions

In this work, we successfully synthesized a large-area thick h-BN film for use as a gate dielectric in electric devices. The h-BN film thicker than the self-limited thickness (10 nm) could be grown by introducing a high-pressure CVD growth mode, where the precursor partial pressure was adjusted beyond a critical pressure (17 mTorr in this work). The atomic and chemical structures of the thin and thick h-BN films were characterized by KPFM, OM, AFM, TEM, SAED, EELS, UV-vis absorption spectroscopy, Raman spectroscopy and XPS. The electrical properties of fabricated MIM (metal/h-BN/metal) and MIS (metal/h-BN/Ge) capacitors revealed that the CVD-grown thickness-controlled h-BN films provided excellent dielectric properties with a high breakdown field and a low leakage current, and the interface quality was excellent, with a reduced interface state density. Our successful CVD growth of thickness-controlled h-BN films was demonstrated by introducing a new high-pressure CVD mode, and its possible applications to electric devices as a gating dielectric were fully confirmed.

## Additional Information

**How to cite this article**: Jang, S. K. *et al.* Synthesis and Characterization of Hexagonal Boron Nitride as a Gate Dielectric. *Sci. Rep.* **6**, 30449; doi: 10.1038/srep30449 (2016).

## Supplementary Material

Supplementary Information
