# Supplementary material for: Synthesis and Characterization of Hexagonal Boron Nitride as a Gate Dielectric
Source: Sci Rep. 2016 Jul 26;6:30449. doi: 10.1038/srep30449 (PMC4960585; doi:10.1038/srep30449)
Supplement: Supplementary Information [file srep30449-s1.pdf]

Supporting Information

Synthesis and Characterization of Hexagonal Boron Nitride as a Gate Dielectric

Sung Kyu Jang, Jiyoun Youn, Young Jae Song and Sungjoo Lee**

SEM images of an h-BN film reveal a continuous and uniform surface on the micrometer scale. The wrinkle on the film resulted from mismatch between the thermal expansion coefficients of the h-BN film and the copper foil during the cooling process (Figure S1a & 1b)[1]. As the film thickness increased beyond a critical thickness (10 nm), the surface morphology became rough with island structures.

Figure S2 shows a representative EELS spectrum obtained from h-BN film. The EELS spectrum displayed sharp K-shell ionization energy levels corresponding to B and N at 185 and 405 eV. The carbon K signal, which would indicate the presence of a residue, was not observed. In each band, the first peak corresponded to the 1s-π antibonding orbital and the second pick corresponded to the 1s-σ antibonding orbital indicative of sp^2-hybridized bonds between the boron and nitrogen atoms.

Figure S3 shows a representative UV-vis absorption spectrum. The absorption peak was located at 201 nm. The optical band gap could be determined from Tauc's equation, $\omega^2\varepsilon = (h\omega - E_g)^2$, where ε, E_g, ω, and h are the optical absorbance, optical band gap, angular frequency, and Planck's constant, respectively[2]. The calculated optical band gap was 6.0 eV, consistent with previous reports[3].

Figure S4a shows the Raman spectra of an h-BN film. A peak corresponding to the E2g mode was observed at 1369 cm^{-2}, consistent with previously reported data[4,5]. Figure S4b and S4c shows the XPS B1s peak centered at 190.4 eV and the N1s peak centered at 397.9 eV, corresponding to the B and N binding energies[6,7], respectively. B and N atoms were present in a 1:1 ratio.

Figure S5 shows the dielectric constant (ε_r) extracted from capacitance measurement. Regardless of the thickness of h-BN film, the dielectric constant is 3-4 which is consistent with previously[7]. The value is comparable with that of h-BN and conventional SiO_2. This independence of film thickness on the dielectric constant indicates that the h-BN/metal interface has a high quality in terms of various defects which can cause dielectric loss[8,9].

An h-BN film with a thickness of less than 10 nm showed a breakdown electric field of above 4 MV/cm. This values are comparable with those of conventional dielectric materials of a few nm thick. The several reported values of breakdown voltage of thin dielectric films are following; Al2O3: 4~5[10], HfO2: 5.4[11], 4[12], SiO2: 4~8[13].

Figure S6 shows the representative data of the I-V characteristics measured at 5 nm and 32 nm thick h-BN films, which were used in calculations of breakdown voltage. All the data were measured under the same conditions. These data of breakdown voltage were statistically analyzed to make a Figure 4b.

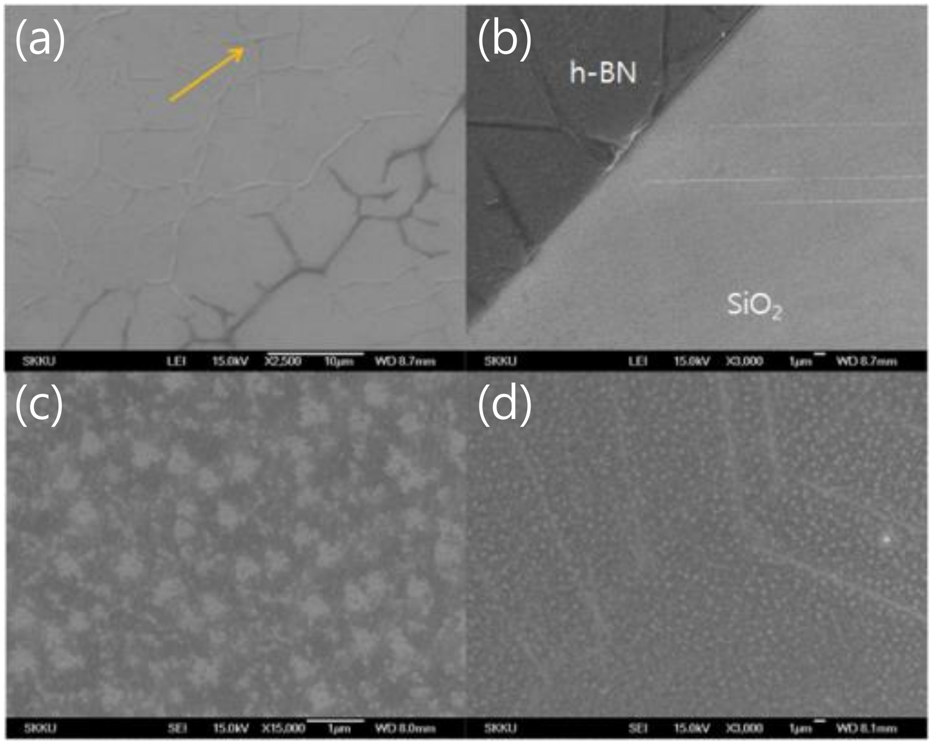

Figure S1. SEM images of the h-BN film transferred to a SiO_2 substrate. (a,b) A typical h-BN film with a thickness of less than 10 nm. The yellow arrow indicates a wrinkle caused by mismatch between the thermal coefficients of h-BN and copper. (c,d) Island structures were observed on the top surface of the h-BN film beyond the critical thickness (>10 nm).

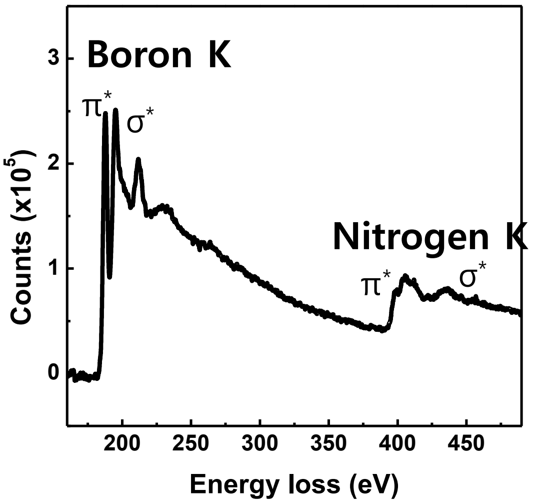

Figure S2. EELS data obtained from the h-BN film.

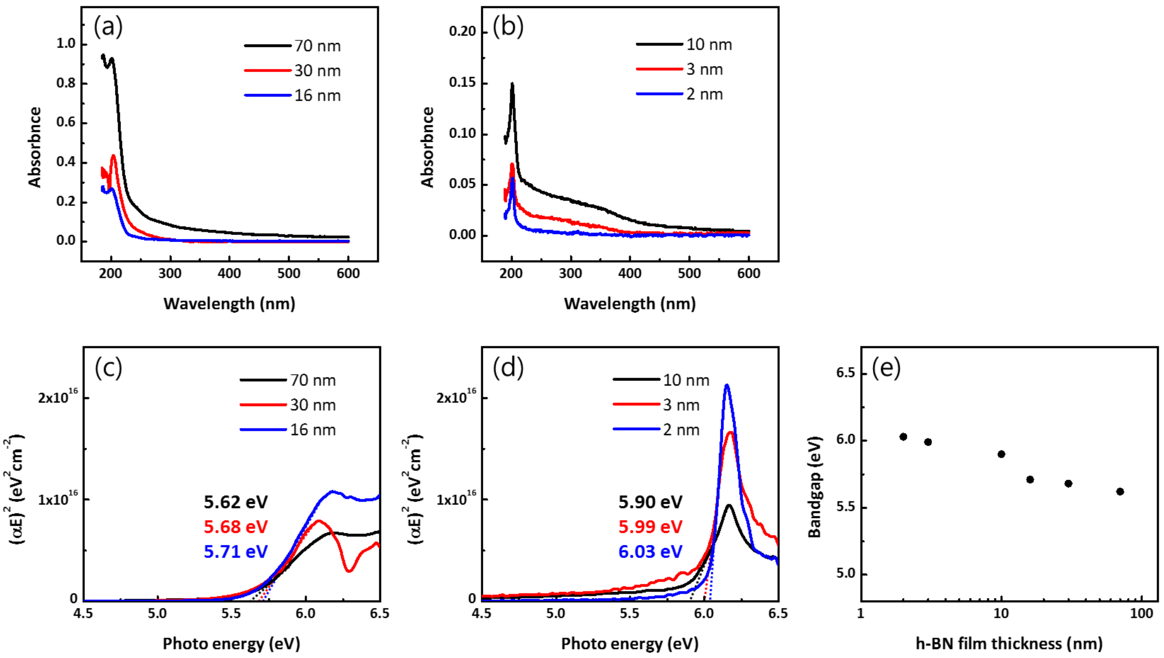

Figure S3. (a, b) UV-visible absorption spectrum and (c, d) corresponding Tauc's plot of h-BN film. (e) The optical band gap calculated by extrapolating the linear trend observed in the Tauc's plot.

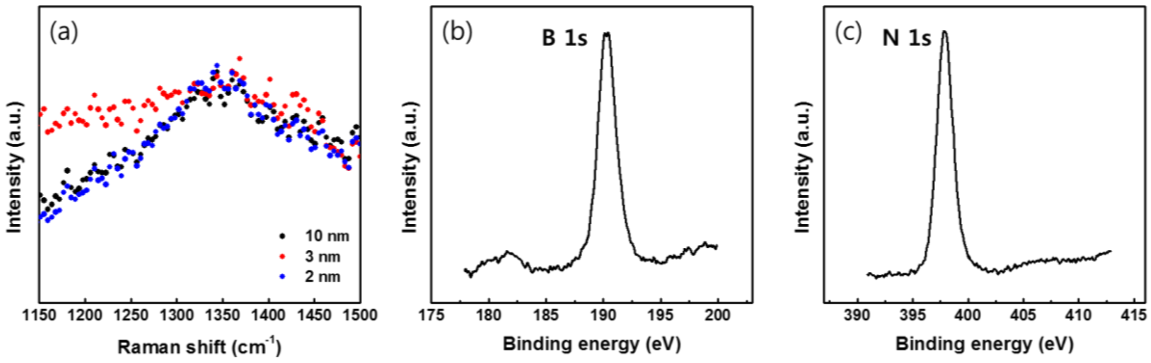

Figure S4. (a) Raman spectra obtained from the h-BN films. (b) B 1s and (c) N 1s XPS spectra obtained from the h-BN film, with peaks at 190.4 eV and 397.9 eV, respectively.

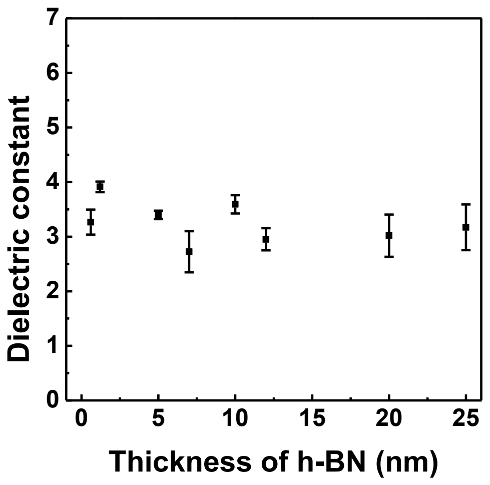

Figure S5. The measured dielectric constant as a function of the h-BN film thickness within the MIM structure.

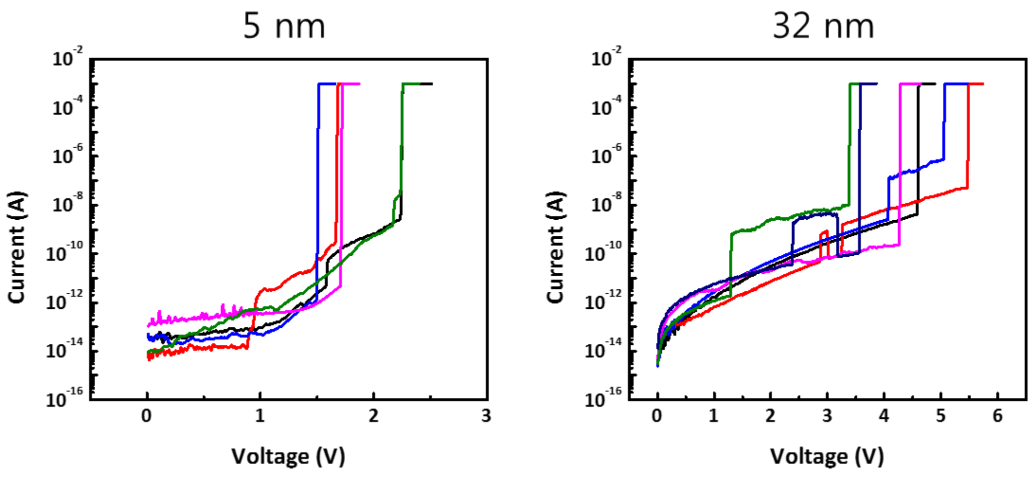

Figure S6. The representative I-V characteristic of 5- and 32nm-thick h-BN films.

Reference

1. Kelly, B. T. The anisotropic thermal expansion of boron nitride. *Philos. Mag.* **32,** 859–867 (1975).
2. Tauc, J. Optical properties and electronic structure of amorphous Ge and Si. *Mater. Res. Bull.* **3,** 37–46 (1968).
3. Kim, G. *et al.* Growth of High-Crystalline, Single-Layer Hexagonal Boron Nitride on Recyclable Platinum Foil. *Nano Lett.* **13,** 1834–1839 (2013).
4. Gao, Y. *et al.* Repeated and Controlled Growth of Monolayer, Bilayer and Few-Layer Hexagonal Boron Nitride on Pt Foils. *ACS Nano* **7,** 5199–5206 (2013).
5. Ci, L. *et al.* Atomic layers of hybridized boron nitride and graphene domains. *Nat. Mater.* **9,** 430–435 (2010).
6. Song, L. *et al.* Large Scale Growth and Characterization of Atomic Hexagonal Boron Nitride Layers. *Nano Lett.* **10,** 3209–3215 (2010).
7. Kim, K. K. *et al.* Synthesis of Monolayer Hexagonal Boron Nitride on Cu Foil Using Chemical Vapor Deposition. *Nano Lett.* **12,** 161–166 (2012).
8. Gupta, S. K. & Srivastava, V. K. Thickness influence on dielectric loss. *J. Phys. Chem. Solids* **37,** 975–977 (1976).
9. Basceri, C., Streiffer, S. K., Kingon, A. I. & Waser, R. The dielectric response as a function of temperature and film thickness of fiber-textured (Ba,Sr)TiO_3 thin films grown by chemical vapor deposition. J. Appl. Phys. 82, 2497 (1997).
10. Yota, J., Shen, H. & Ramanathan, R. Characterization of atomic layer deposition HfO_2, Al_2O_3, and plasma-enhanced chemical vapor deposition Si_3N_4 as metal–insulator–metal capacitor dielectric for GaAs HBT technology. *J. Vac. Sci. Technol. A Vacuum, Surfaces, Film.* **31,** 01A134 (2013).
11. Kolodzey, J. *et al.* Electrical conduction and dielectric breakdown in aluminum oxide insulators on silicon. *IEEE Trans. Electron Devices* **47,** 121–128 (2000).
12. Farcy, A., Carpentier, J.-F., Thomas, M., Torres, J. & Ancey, P. Integration of high-performance RF passive modules (MIM capacitors and inductors) in advanced BEOL. *Microelectron. Eng.* **85,** 1940–1946 (2008).
13. Chang, Y. C. *et al.* Atomic-layer-deposited HfO_2 on $In_{0.53}Ga_{0.47}As$: Passivation and energy-band parameters. *Appl. Phys. Lett.* **92,** 072901 (2008).
